# Soil carbon mineralization in response to nitrogen enrichment in surface and subsurface layers in two land use types

**DOI:** 10.7717/peerj.7130

**Published:** 2019-07-08

**Authors:** Nazia Perveen, Mariam Ayub, Tanvir Shahzad, Muhammad Rashid Siddiq, Muhammad Sohail Memon, Sébastien Barot, Hamid Saeed, Ming Xu

**Affiliations:** 1 Key Laboratory of Geospatial Technology for the Middle and Lower Yellow River Regions, College of Environment and Planning, Henan University, Kaifeng, China; 2 Department of Environmental Sciences, Forman Christian College (A Chartered University), Lahore, Pakistan; 3 Department of Environmental Sciences & Engineering, Government College University Faisalabad, Faisalabad, Pakistan; 4 Institut für Biowissenschaften, Technische Universität Bergakademie Freiberg, Freiberg, Germany; 5 College of Engineering, Nanjing Agricultural University, Nanjing, Jiangsu, China; 6 IEES-Paris (IRD, CNRS, UPMC, INRA, UPEC), 4 place Jussieu, Paris, France; 7 Faculty of Agricultural Engineering, Sindh Agriculture University, Tandojam, Pakistan

**Keywords:** Soil c mineralization, Nitrogen enrichment, Land use types, C sequestration, Subsurface soil C

## Abstract

Atmospheric nitrogen (N) deposition increases N availability in soils, with consequences affecting the decomposition of soil carbon (C). The impacts of increasing N availability on surface soil C dynamics are well studied. However, subsurface soils have been paid less attention although more than 50% soil C stock is present below this depth (below 20 cm). This study was designed to investigate the response of surface (0–20 cm) and subsurface (20–40 cm and 40–60 cm) C dynamics to 0 (0 kg N ha^−1^), low (70 kg N ha^−1^) and high (120 kg N ha^−1^) levels of N enrichment. The soils were sampled from a cropland and a grass lawn and incubated at 25 °C and 60% water holding capacity for 45 days. Results showed that N enrichment significantly decreased soil C mineralization (Rs) in all the three soil layers in the two studied sites (*p* < 0.05). The mineralization per unit soil organic carbon (SOC) increased with profile depth in both soils, indicating the higher decomposability of soil C down the soil profile. Moreover, high N level exhibited stronger suppression effect on Rs than low N level. Rs was significantly and positively correlated with microbial biomass carbon explaining 80% of variation in Rs. Overall; these results suggest that N enrichment may increase C sequestration both in surface and subsurface layers, by reducing C loss through mineralization.

## Introduction

Nitrogen (N) is one of the most important elements in nature. However, human activities such as fossil fuel combustion, food and energy production and land use change have greatly accelerated the atmospheric deposition of reactive N to the biosphere (Vitousek et al., 1997; Galloway et al., 2004). Moreover, global N deposition rate is projected to increase by a factor of 2.5 from its current levels by the end of this century and it will continue increasing especially in eastern and southern Asia (Galloway et al., 2004; Lamarque et al., 2005; Denman et al., 2007). This N enrichment has negative impacts on ecosystem functioning, biotic diversity and terrestrial carbon (C) cycling (Vitousek et al., 1997; Bobbink, Hornung & Roelofs, 1998; Stevens et al., 2004; Perveen et al., 2014) with consequent feedbacks on global climate change.

Several studies have investigated the impacts of N enrichment on soil CO_2_ emission by manipulating the availability of mineral N to soil. However, most studies have focused exclusively on the surface 20 cm of soil (Fontaine et al., 2011; Yan et al., 2016; Zhu et al., 2016) despite that more than 50% of the total SOC is stored in the subsurface soils (below 20 cm) (Jobbágy & Jackson, 2000). This large pool of deep soil C has high potential to alter the global C cycle and future climate if its mineralization by soil microorganisms is stimulated in response to global changes (e.g., elevated atmospheric N deposition, land use change, deep rooted cropping etc.). Despite this importance, very few studies have investigated the response of subsurface C dynamics to N enrichment (Fierer et al., 2003; Medlyn et al., 2015; Kaneez-e Batool et al., 2016), although it has started gaining considerable attention recently (Wordell-Dietrich, Don & Helfrich, 2017; Shahzad et al., 2018; Shahzad et al., 2019). The reasons for neglecting subsoil C in soil organic matter studies have been; (1) the assumption that subsoil C dynamics are similar to those of surface soil C albeit with lower turnover rate owing to poor quality of subsoil C, (2) the age of subsoil C in centuries to millennia years, discovered thanks to ^14^C dating, led to an assumption that the subsoil C is quasi-permanent and will not matter in climate change scenarios (Jenkinson, Poulton & Bryant, 2008; Salomé et al., 2010). However, recent investigations have challenged these assumptions. For example, furnishing fresh C in subsoil layers induced mineralization of millennia old subsoil C (Fontaine et al., 2007; Shahzad et al., 2018; Perveen et al., 2019). Similarly, physical disturbance or drying-wetting cycles imposed on subsoil samples led to release of centuries to thousands of years old subsoil C (Ewing et al., 2006; Schimel et al., 2011). These studies underline that the subsoil C cycling is liable to change quickly in response to environmental disturbances. Therefore, it is important to understand the effect of N deposition on subsoil C dynamics as well in order to devise suitable policies for C sequestration.

Past studies examining the responses of soil C mineralization (Rs) to experimental N enrichment remain inconsistent both in terms of magnitude and direction. Some studies have shown that N enrichment increased the release of soil CO_2_ in grasslands (Xu et al., 2004; Han et al., 2012; Zhang et al., 2014; Zhou et al., 2014), forests and croplands (Bowden et al., 2004; Zhou et al., 2014; Wang et al., 2015). In contrast, other studies found a reduction in soil CO_2_ release in forest and grassland soils in response to N enrichment (Janssens et al., 2010; Lu et al., 2011; Zhou et al., 2013); (Sun et al., 2014; Riggs & Hobbie, 2016). It has also been reported that increased N enrichment may lead to the stabilization of the added organic matter (Fontaine et al., 2011; Shahzad et al., 2012). Moreover, non-significant changes in soil CO_2_ release in response to N enrichment have also been reported (Lu et al., 2011). These contradictory results requires further investigations on this topic to deepen our current understanding of the effects of N enrichment on terrestrial C cycling.

The objective of this study was to investigate the effect of N enrichment on soil C mineralization (Rs) in surface (0–20 cm) and subsurface (20–40 cm and 40–60 cm) layers of two land use systems. We performed an incubation experiment using soils collected from a long-term wheat-maize rotation field (>17 years) and grass lawn (>50 years). The soils were amended with two levels of mineral N whereas unamended soils were used as controls. We expected different responses to N enrichment in two soils because of their different land uses as well as physico-chemical properties.

## Materials and Methods

### Soil sampling and analyses

In May 2016, soil samples were collected from 0–20 cm, 20–40 cm and 40–60 cm layers from the wheat field of University of Agriculture Faisalabad, Pakistan (31°23′41″N, 73°3′0″E) and the grass lawn of Forman Christian College Lahore, Pakistan (31°15′45″N, 74°0′1″E). The dead and live vegetation and stones were removed from the soil samples by hand. All the soil samples were homogenized, sieved through a 2-mm mesh and analyzed for soil organic C (SOC), pH, water holding capacity (WHC) and texture (sand, silt, clay) (Table 1).

**Table 1:** Physical and chemical properties of the soils used in incubation experiment.

	pH	SOC	Total N	C/N	WHC	Sand	Silt	Clay	Textural class
		g C kg^−1^ soil	g N kg^−1^ soil		(%)	(%)	(%)	(%)	
**0–20 cm**									
Cropland	8.26a	3.46b	0.29b	11.9a	38.74b	56.5a	20.6a	22.9b	Sandy clay loam
Grass lawn	8.09a	7.47a	0.62a	12.0a	49.22a	53.9b	21.0a	25.0a	Sandy clay loam
**20–40 cm**									
Cropland	8.82a	1.37b	0.27b	5.1a	33.46b	52.9a	21.8a	25.3b	Sandy clay loam
Grass lawn	8.40b	4.12a	0.82a	5.0a	44.96a	53.1a	17.4b	29.4a	Sandy clay loam
**40–60 cm**									
Cropland	8.33a	0.36b	0.05b	7.2a	33.89b	50.8a	18.7a	30.5a	Sandy clay loam
Grass lawn	8.39a	2.43a	0.35a	6.9a	46.69a	51.2a	18.2a	30.5a	Sandy clay loam

**Notes.**

SOC soil organic carbon Total N total soil nitrogen WHC water holding capacity

Different letters after the numerical values indicate significant differences between two studied land uses.

Soil pH was analyzed in a 1:5, soil: water ratio using a pre-calibrated pH meter. Soil water holding capacity (WHC) was determined following the method of Jarrell (Jarrell et al., 1999). The SOC was measured according to Walkley–Black (Walkley & Black, 1934). Soil texture was measured following Bouyoucos hydrometer method (Gee & Bauder, 1979). Soil samples were pre-incubated for two weeks after adjusting the WHC at 60%.

### Incubation experiment

The incubation experiment included three treatments with three replicates: soil amended with 0 kg N ha^−1^ (control), 70 kg N ha^−1^ (Low N, LN) and 120 (High N, HN) kg N ha^−1^, respectively. 20 g (on the oven-dried basis) of soil was placed in 500 ml plastic jars. The NH_4_NO_3_ dissolved in distilled water was added to develop the two N treatments and mixed. The control samples were also mixed to apply the same physical disturbance. The soil moisture was adjusted to 60% water holding capacity (WHC). Two 20-mL glass vials were placed in the plastic jars, one containing 0.05 M NaOH to trap CO_2_ released from soil C mineralization and another with 10 mL distilled water to avoid soil dryness. The blanks were also performed by placing the glass vials of NaOH and distilled water only. The jars were closed with air-tight caps and placed in an incubator at 25 °C for 45 days. The sampling of NaOH was performed at day 1, 3, 7, 15, 21, 28, 35 and 45, respectively. The concentration of CO_2_ in NaOH was precipitated with 0.5 M BaCl_2_ followed by titration against 0.1 M HCl using phenolphthalein as indicator (Isermeyer, 1952; Jaggi, 1976). At each gas sampling day, glass vial containing NaOH was replaced and water loss from soil was supplemented after weighing the sample.

### Microbial biomass extraction

The microbial biomass carbon (MBC) was determined by the fumigation extraction technique (Sparling & West, 1988). For each destructive sampling, 5 g of soil was extracted with 20 mL of 30 mM K_2_SO_4_ and shacked for 1 h (non-fumigated sample). Another 5 g sample was fumigated with ethanol-free chloroform for 24 h in a glass desiccator. Chloroform was removed from the soil by ventilation, and the soil was immediately extracted with 20 mL of 30 mM K_2_SO_4_. The K_2_SO_4_ extracts were filtered (0.45 m) and then lyophilized. The recovered crystals were stored until analysis of C content. The microbial biomass was calculated as the difference of organic C between the fumigated and non-fumigated extracts using a conversion factor of 0.45 (Brookes et al., 1985).

### Statistical analyses

The impact of N enrichment, land use and profile layer on soil C mineralization (Rs) and MBC was evaluated using one-way ANOVA and the least significant test (LSD) was used to compare the mean differences among N treatments. The difference in soil properties and MBC between two land uses and different layers were also evaluated using one-way ANOVA. The relationships between MBC, soil C content and Rs were evaluated using Pearson’s correlation. All statistical analyses were performed with R 3.4.3 (R Core Team, 2017).

## Results

### Soil properties

The physical and chemical properties of the soils are shown in Table 1. Soil pH ranged from 8.09 to 8.82, however, no significant difference was found between two soils except in 20–40 cm layer where pH of cropland soil was higher than of grass lawn. Water holding capacity was significantly higher in grass lawn compared to crop land in the whole soil profile (*p* < 0.05). The SOC content, initial MBC and total N decreased with the profile depth in both soils, and were significantly higher in grass lawn than those in cropland (*p* < 0.05). The SOC content in 0–20 cm soil layer of grass lawn was 1.81 and 3.08 times higher than that in 20–40 cm and 40–60 cm layers of the same soil, respectively. The SOC content in 0–20 cm layer of cropland was 2.53 and 9.50 times higher than that in 20–40 cm and 40–60 cm layers, respectively. The initial MBC in 0–20 cm soil layer of grass lawn was 1.22 and 1.44 times higher than that in 20–40 cm and 40–60 cm layers, respectively. The initial MBC in 0–20 cm layer of cropland was 1.15 and 1.72 times higher than that in 20–40 cm and 40–60 cm layers, respectively. The total C/N ratio was apparently similar between two soils along the whole profile (*p* > 0.05). Both soils were characterized by a sandy clay loam texture (Table 1).

### Soil C mineralization

**Figure 1:**
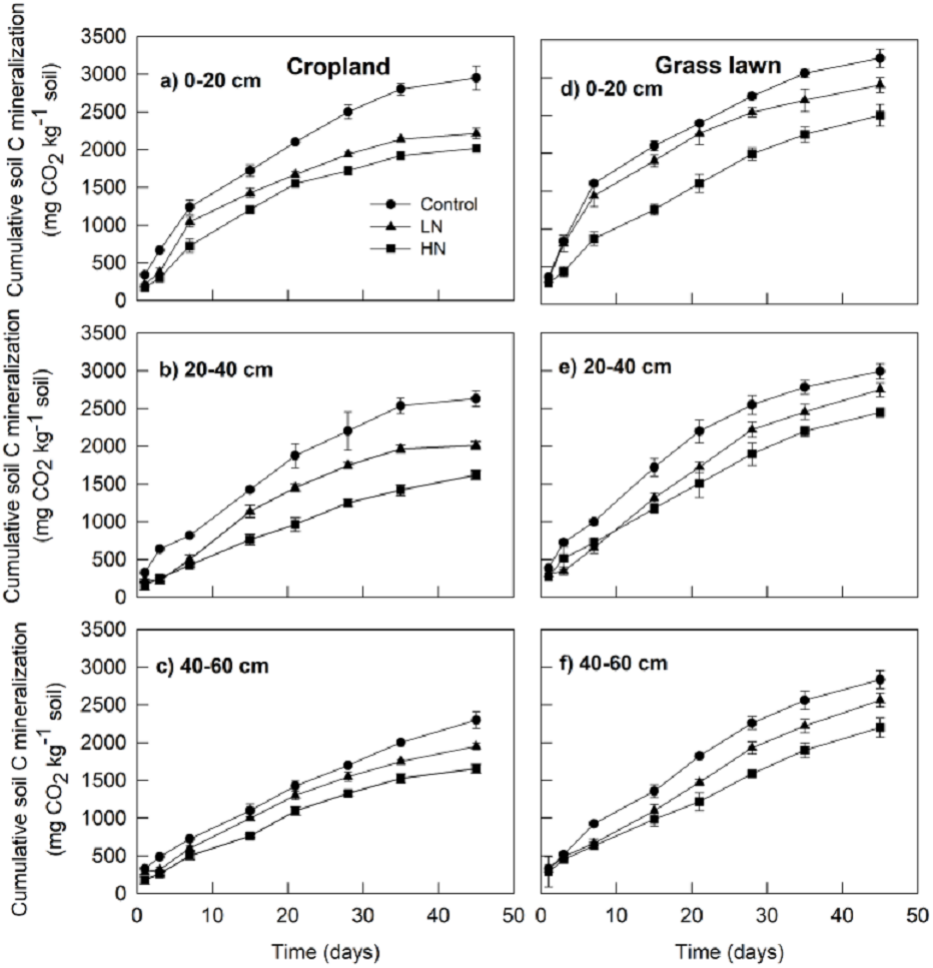
Effects of nitrogen (N) addition on cumulative soil C mineralization (mg C-CO_2_ kg^−1^ soil). Values are given as mean ± standard error. Control, no N addition; LN, low level N addition; HN, high level N addition. Cropland: (A) 0–20 cm, (B) 20–40 cm, (C) 40–60 cm. Grass lawn: (D) 0–20 cm, (E) 20–40 cm, (F) 40–60 cm.

The results showed that cumulative soil C mineralization, Rs (mg CO_2_ kg^−1^ soil) from different layers in grass lawn was significantly higher than corresponding values in cropland during the 45-days incubation period (Fig. 1). Rs from control treatment in cropland was significantly different among the three layers (*p* < 0.05), decreasing with the soil profile. In grass lawn, Rs under control treatment was significantly higher in 0–20 cm layer than that in the 20–40 and 40–60 cm layers (*p* < 0.05); however, there was no apparent difference between the two latter layers (*p* > 0.05).

The N enrichment (LN and HN) significantly decreased Rs compared to correspondent controls in the three layers of both soils (*p* < 0.05) (Fig. 1). However, cumulative CO_2_ emission under LN was significantly higher than under HN for all soils (*p* < 0.05). In cropland, Rs under HN was decreased by 10%, 24%, and 18% in the 0–20, 20–40 and 40–60 cm layer soils, respectively, compared to LN. In grass lawn, the decrease in Rs under HN was 16%, 12%, and 16%, respectively, in 0–20, 20–40 and 40–60 cm soil layers relative to LN.

### Microbial biomass carbon (MBC)

**Figure 2:**
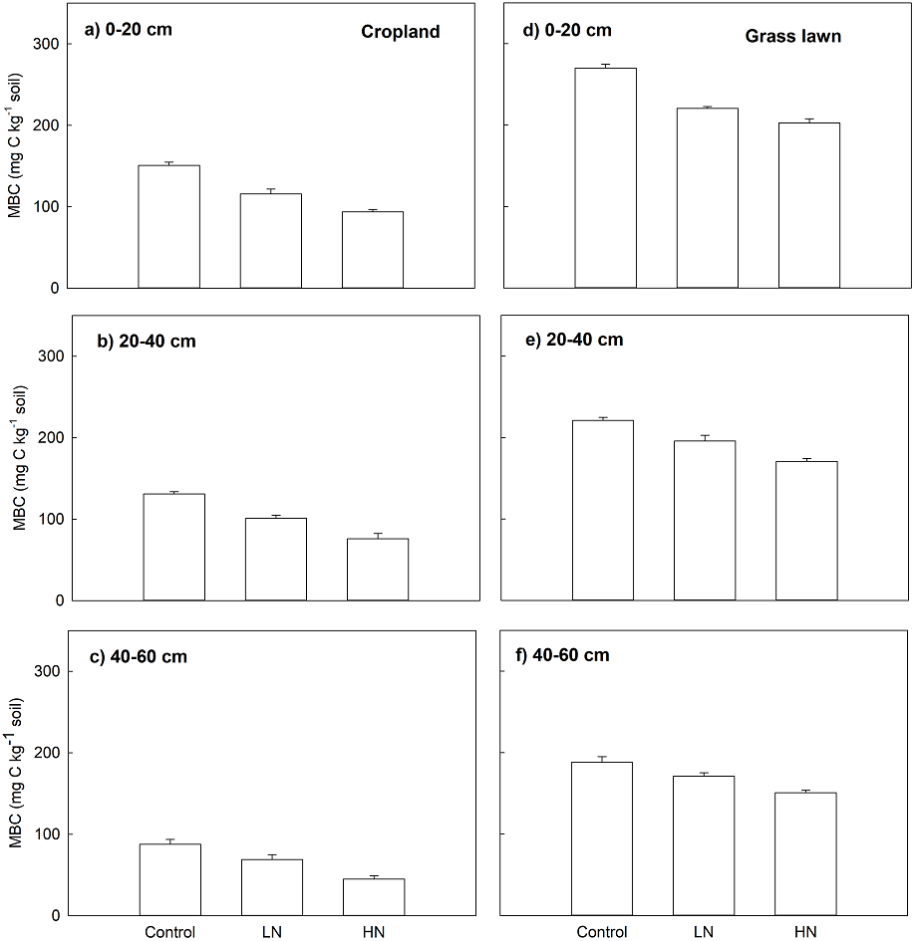
Effects of nitrogen (N) addition on microbial biomass carbon, MBC (mg C kg^−1^ soil). Values are given as mean ± standard error. Control, no N addition; LN, low level N addition; HN, high level N addition. Cropland: (A) 0–20 cm, (B) 20–40 cm, (C) 40–60 cm. Grass lawn: (D) 0–20 cm, (E) 20–40 cm, (F) 40–60 cm.

Nitrogen enrichment (LN and HN) also significantly decreased MBC (mg C kg^−1^ soil), MBC under LN and HN treatments in two studied soils was significantly lower compared to their correspondent controls along the whole profile (*p* < 0.05, Fig. 2). Similar to Rs, the decrease in MBC under HN was more intense than that of under LN. In cropland, the decrease ratio in MBC under HN was 23%, 33% and 53%, respectively, in 0–20, 20–40 and 40–60 cm soil layers relative to LN (Fig. 2). In grass lawn, MBC under HN was decreased by 9%, 15%, and 13% in the 0–20, 20–40 and 40–60 cm soil layers, respectively, compared to LN (Fig. 2).

### Soil C mineralization related to soil properties

**Figure 3:**
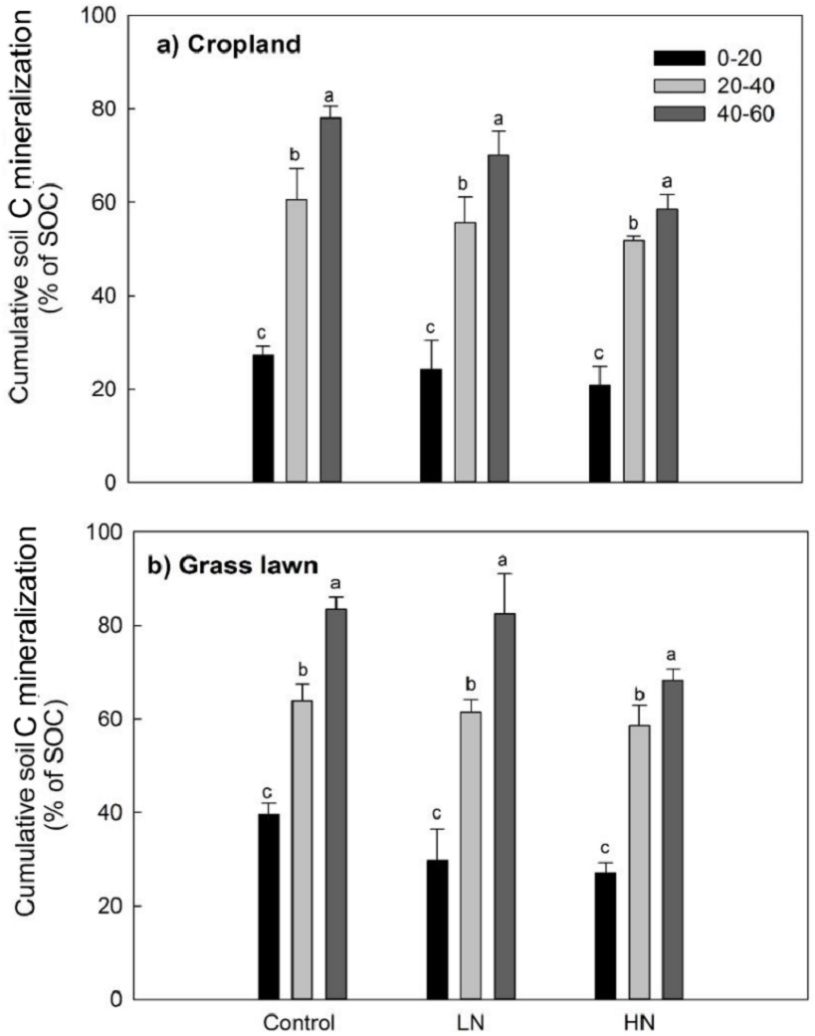
Cumulative soil C mineralization (% of SOC) in response to the nitrogen treatments along the soil profile (*n* = 3). Values are given as mean ± standard error. Control, no N addition; LN, low level N addition; HN, high level N addition. (A) Cropland. (B) Grass lawn.

**Figure 4:**
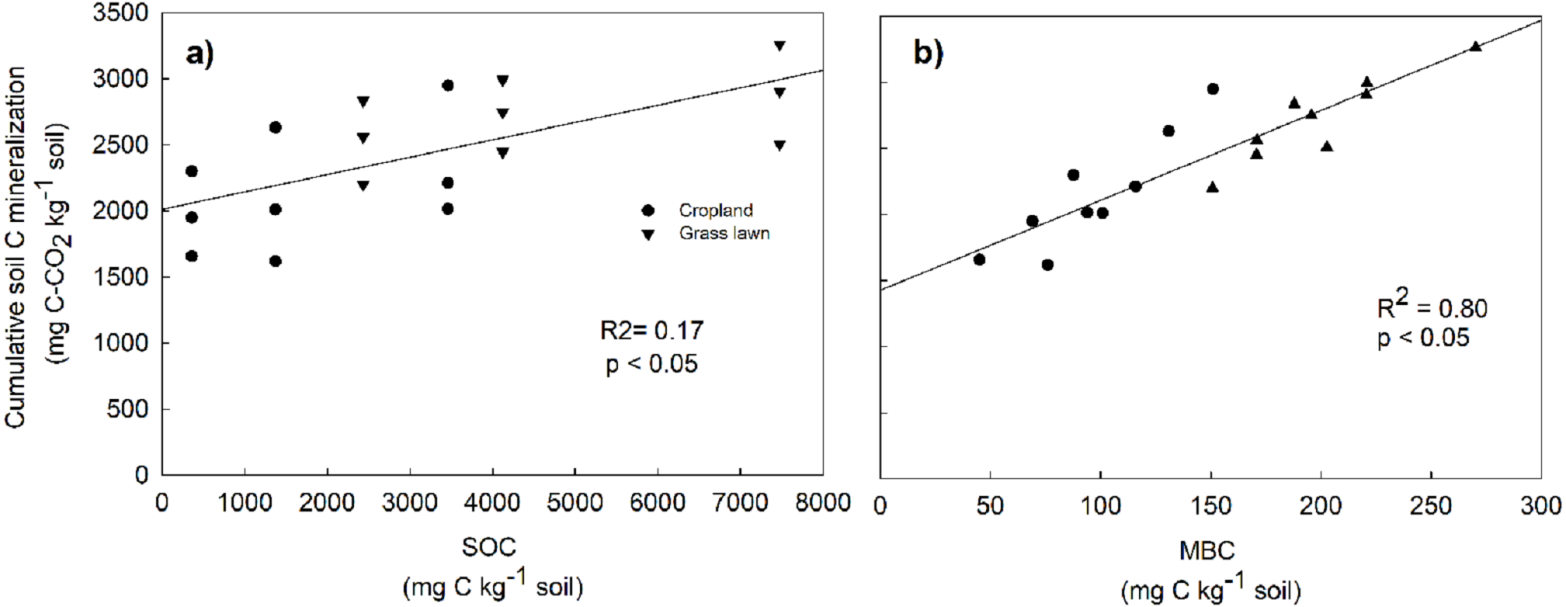
Relationship between cumulative soil C mineralization and soil organic carbon (SOC) (A), and microbial biomass carbon (MBC) (B), across the two land use types and three soil layers. The correlations were calculated for individual values.

Rs as percentage of SOC was presented to give an idea about the microbial activities based on the initial substrate availability and the results showed that it increased with the profile depth in control and N amended treatments in studied sites (Fig. 3). Further, Rs was significantly and positively correlated with both SOC (*R*^2^ = 0.52) and MBC (*R*^2^ = 0.80) (*p* < 0.05, Fig. 4). The change in Rs relative to the control soil in response to the N addition showed that Rs was higher in subsurface than in surface layers in two studied soils (Table 2).

## Discussion

In this study, N addition to soils significantly reduced soil C mineralization (Rs) in the surface and subsurface layers, favoring soil C sequestration in both land use types (*p* < 0.05, Fig. 1). The reduction of Rs was not of the same order of magnitude at the two N application levels being more intense at HN than at LN (*p* < 0.05). Consistent to our findings, a decrease of soil CO_2_ emission under N addition has been observed in some previous studies conducted in cropland and grassland ecosystems (Al-Kaisi, Kruse & Sawyer, 2008; Zhu et al., 2016; Riggs et al., 2015; Riggs & Hobbie, 2016; Guo et al., 2017; Wei et al., 2018). This result suggests that the soil microorganisms were decomposing SOC for C as well as mineral N prior to availability of external mineral N. However, the availability of mineral N in excess might have triggered microorganisms to limit their expenditures on SOC-decomposing enzymes for they could assimilate the mineral N thereby leading to reduction in Rs (Fontaine et al., 2011; Shahzad et al., 2012; Kaneez-e Batool et al., 2016).

The negative effect of N application was not only limited to surface layers. It also suppressed C mineralization in the subsoil layers of both soils indicating that the subsoil microorganisms adopted the same strategy of using easily available mineral N instead of keeping on mineralization SOC. Previous studies have also found suppressed SOC mineralization in subsoil layers in response to mineral N addition (Kaneez-e Batool et al., 2016).

In addition to inducing reduced soil C mineralization, the N application significantly decreased soil microbial biomass (Fig. 2). This result corresponds to a well-known meta-analysis conducted for a range of ecosystems whereby it was found that N application reduces soil microbial biomass when C is limited (Treseder, 2008). Our result shows that the increased N availability imposes C limitation on the soil microbes where they are forced to downregulate their growth. Moreover, both the soils used in this study are poor in phosphorus. Similarly, in the presence of labile C sources, the microbes can dig SOC and mineralize P for their growth (Amador & Jones, 1993; Spohn & Kuzyakov, 2013). However, given that we did not add any C source, addition of N could have further stressed microbes in terms of growth resulting into reduced soil microbial biomass.

The soil C mineralization was lower in cropland than grass lawn both in control and N added soils (Fig. 1). This difference occurred because different vegetation types usually lead to a varying quantity and quality of C input and nutrient availability in the soil (Liu & Greaver, 2010; Yang & Zhu, 2015). Moreover, the tillage practices in cropland disturb the soil structure by breaking the soil aggregates and lower SOC content (Table 1) which constrains substrate supply to microorganisms and limits their biomass (Fig. 2) and degradation activity (Chen et al., 2014a; Chen et al., 2014b). In contrast, the N demand rate in the grass lawn was lower than that in the croplands as the grass lawn was not being harvested and little biomass was produced and the senescent biomass was entirely returned to the soil. Thus, the vegetation type could play an important role in regulating terrestrial C cycle feedback to climate change under N deposition.

**Table 2:** The change in soil C mineralization (Rs) relative to the control soil in response to the N addition treatments. Values are means of three replicates and the variances are the standard error of means and are given in %.

		Soil depth (cm)	
Land use	Treatment	0–20	20–40	40–60
Cropland	LN	−25.0 ± 1.1	−23.6 ± 0.9	−15.2 ± 0.9
	HN	−31.7 ± 0.6	−38.4 ± 1.1	−28.0 ± 1.2
Grass lawn	LN	−10.9 ± 0.6	−12.4 ± 2.9	−13.5 ± 2.7
	HN	−23.2 ± 2.1	−18.2 ± 0.9	−22.4 ± 2.1

**Notes.**

LN low level N addition HN high level N addition.

Variations in soil C decomposition normalized for native SOC content has been previously used as an indicator in variations of the decomposability of SOM, the two being positively related (Salomé et al., 2010; Chen et al., 2014a; Chen et al., 2014b). The results of this study showed that the proportion of total C mineralized was significantly higher for each successive subsurface layer compared to the surface layer for two studied soils (Table 2, Fig. 3). This observation was true both for N added treatments and control soils. This result indicates that the decomposability of the organic matter available to soil microbes did not decrease with depth but rather increased in contrast to what has been found in some previous studies (Lomander, Katterer & Andren, 1998; Fierer et al., 2003). Moreover, this result suggests that subsurface soil microorganisms are as active as surface microorganisms except that they are limited by substrate availability, supporting some previous findings (Fontaine et al., 2007; Stone & Plante, 2015).

As with any experimental work, our study is not without certain limitations. Although, our study reveals that different soil layers may response similarly to N addition in two land use types, these results should not be compared to *in-situ* conditions since moisture and temperature conditions may vary along the soil profile. Moreover, it is important for future studies to consider the autotrophic and heterotrophic components of soil respiration individually while evaluating the response of different soil layers to N availability.

## Conclusion

We found that N addition reduced C mineralization along the whole soil profile in the studied land uses via reduction in microbial biomass carbon. However, the intensity of the reduction depends on the concentration of applied N like high N amendment had a stronger effect on suppression of Rs than low N amendment. Therefore, high levels of N additions to soils through atmospheric N deposition, N fertilization and/or agricultural runoff could increase C sequestration in soils.

##  Supplemental Information

10.7717/peerj.7130/supp-1 Data S1 Soil properties of studied sites Each soil depth has 3 replicates.

10.7717/peerj.7130/supp-2 Data S2 Soil C mineralization of studied sites Each soil depth has 3 replicates.

## Additional Information and Declarations

### Data Availability

The following information was supplied regarding data availability: The raw data of soil properties and the raw measurements of soil respiration are available as Supplemental Files. This data was used for all statistical analyses.
